# Unusual Etiology for a Chronic Subdural Hematoma: Whole-Body Vibration Machine

**DOI:** 10.7759/cureus.21359

**Published:** 2022-01-18

**Authors:** Orlando De Jesus, Juan López Fontanet

**Affiliations:** 1 Neurosurgery, University of Puerto Rico, Medical Sciences Campus, San Juan, PRI

**Keywords:** vibration machine, trauma, subdural hematoma, geriatric, etiology, chronic, arachnoid cyst

## Abstract

A chronic subdural hematoma is more common in the elderly, particularly after a minor trauma sustained several weeks before the diagnosis. The use of a whole-body vibration machine had not been reported as an etiology. We report an 80-year-old male patient without a history of head or body trauma who developed a bilateral chronic subdural hematoma and required surgery to drain the hematoma. Four weeks before the diagnosis, he purchased and used a whole-body vibration machine. The first time he used the whole-body vibration machine, he felt his brain vibrating and rotating and could not tolerate more than two minutes. The event was so annoying that he did not use it again. In this patient, using a whole-body vibration machine may have led to the formation of the chronic subdural hematoma.

## Introduction

A chronic subdural hematoma (CSDH) is a collection of blood under the dura mater compressing the brain with several weeks of development. It is more common in the elderly, particularly after a minor trauma sustained several weeks before the diagnosis. Although trauma is a significant factor for developing a CSDH, up to 50% of the patients do not have a history of direct head injury [1]. Other predisposing factors include anticoagulation, bleeding diathesis, alcoholism, epilepsy, removal of cerebrospinal fluid, and dialysis [1]. An intracranial arachnoid cyst is also a predisposing factor in children and younger patients after minor head trauma [2-4]. Development of a CSDH after a roller coaster ride had been reported in the literature [5-8]. The acceleration and rotational forces that the head sustains during the roller coaster ride may cause the formation of the hematoma. These forces can produce the rupture of bridging dural veins, with bleeding into the subdural space. If the accumulated subdural blood does not break down and is eventually absorbed, the hematoma gradually enlarges and forms a CSDH. The use of a whole-body vibration machine had not been reported as an etiology for a CSDH. We present a patient who used a whole-body vibration machine four weeks before his diagnosis of a bilateral CSDH.

## Case presentation

**Figure 1:**
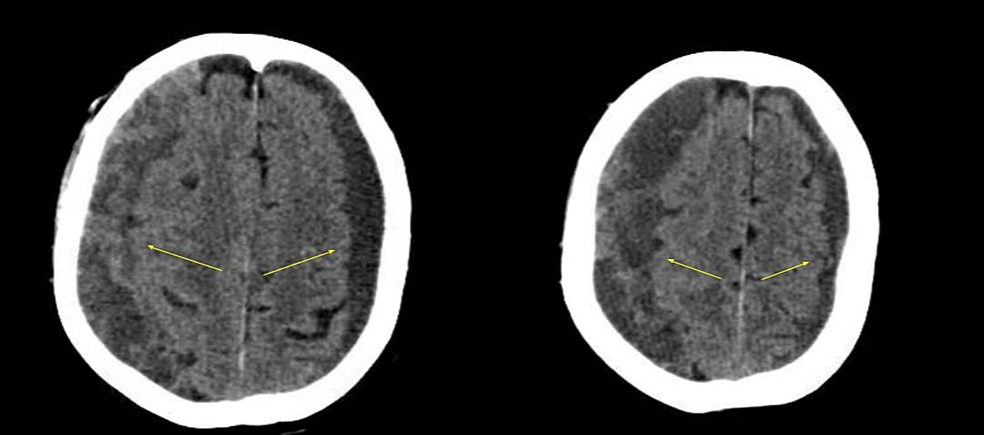
Head axial computed tomographic scan showing a bilateral chronic subdural hematoma (yellow arrows).

A healthy 80-year-old male patient suffered a faint episode while watching television, rapidly recovering in approximately ten seconds. His wife encouraged him to go to the primary physician, who found him with stable vital signs and a normal physical examination. He was sent home to set up a syncope workup. The following morning, he was disoriented, rapidly progressing to lethargy, and was promptly taken to the hospital for evaluation. On the physical examination, he localized bilaterally to painful stimuli but did not follow commands. His verbal response only produced incoherent words. His wife denied any history of head trauma or seizures. He was not using any antiplatelet or anticoagulation medications. Past medical history was relevant for the presence of a sellar/suprasellar arachnoid cyst operated 12 years before using a transsphenoidal approach. A head computed tomographic (CT) scan was performed, which showed a bilateral CSDH (Figure 1).

**Figure 2:**
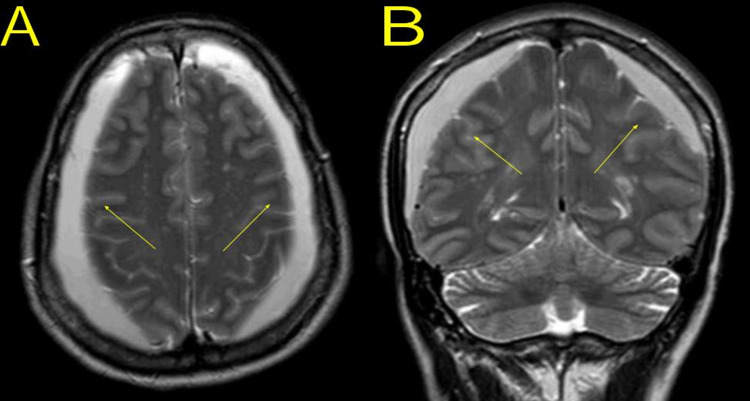
Brain magnetic resonance imaging showing the bilateral chronic subdural hematoma (yellow arrows): (A) axial T2-weighted image, (B) coronal T2-weighted image.

Brain magnetic resonance imaging (MRI) was also done to evaluate for other intracranial anomalies; however, it only showed the bilateral CSDH and the previously known sellar/suprasellar arachnoid cyst without evidence of intracystic bleeding (Figure 2).

He was immediately operated on with bilateral burr holes at the frontal and parietal areas. Two hours after the surgery, he followed commands and maintained a conversation. The patient and his wife denied a history of head or body trauma during the previous six months. Upon further questioning the patient about his routine activities predisposing him to develop a chronic hematoma, he recalled purchasing and using a whole-body vibration machine four weeks before the operation. The first time he used the whole-body vibration machine, he felt his brain vibrating and rotating and could not tolerate more than two minutes. He recalled developing a moderate intensity headache a few minutes later; however, he did not seek medical evaluation as the headache rapidly resolved after taking a non-steroidal anti-inflammatory drug. The episode was so annoying that he did not use it again. He was discharged home two days after the operation without any residual deficit.

**Figure 3:**
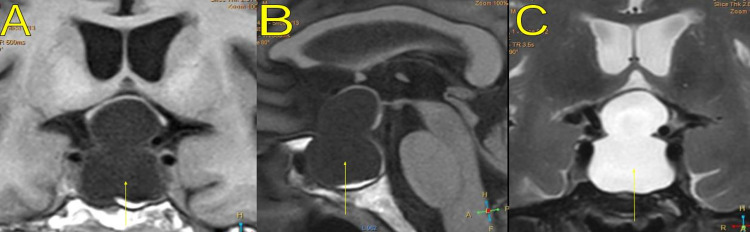
Brain magnetic resonance imaging without contrast performed 16 months later, showing a large sellar/suprasellar arachnoid cyst (yellow arrows), which was stable in size compared to all prior studies: (A) coronal T1-weighted image, (B) sagittal T1-weighted image, (C) coronal T2-weighted image.

A head CT scan performed three months after the operation showed the resolution of the chronic bilateral hematoma. Neurological examination was normal. Visual field examination showed no deficits. A brain MRI was performed 12 months later, which showed that the large sellar/suprasellar arachnoid cyst remained stable compared to prior studies (Figure 3).

## Discussion

Cerebral atrophy and increased venous fragility in the elderly are major predisposing factors for developing a CSDH [1]. As a consequence of cerebral atrophy, the space between the brain and the skull enlarges, causing the dural bridging veins to stretch. Even minor head trauma or falls can cause these veins to bleed as there is greater brain movement within the skull. After a hemorrhage occurs in the subdural space, the outer surface of the hematoma is initially covered by a thin layer of fibrin and fibroblasts, which forms an outer membrane [1]. Liquefaction of the hematoma subsequently occurs due to the presence of phagocytes. Neovascularization of the outer membrane produces new microhemorrhages and allows more fluid to enter the hematoma, gradually enlarging the collection. Ultimately, an inner membrane is also formed just over the cerebral cortex. Clinical symptoms vary from a simple headache to a coma as the hematoma gradually compresses the brain. Most patients with a large and symptomatic CSDH require surgical treatment.

After reviewing the literature, we could not find a report of a CSDH associated with the use of a whole-body vibration machine. Park et al. reported a patient who developed a bilateral CSDH after using a vibrating belt machine on the head for 20 days [9]. The authors attributed the subdural bleeding to the acceleration and deceleration of the brain induced by the vibration [9]. Acceleration and rotational forces experienced after a roller coaster ride had been attributed as a factor for developing a CSDH [5-8]. The head and brain movements while using a whole-body vibration machine for a few minutes may be comparable to a roller coaster ride. These movements may have led to the chronic subdural hematoma in our patient.

Patients with arachnoid cysts are prone to develop a CSDH as the cerebral veins are stretched and predisposed to rupture after a trauma. A CSDH occurs more frequently with temporal arachnoid cysts and occasionally with convexity arachnoid cysts. Suprasellar arachnoid cysts have not been associated with a CSDH over the cerebral convexity. The literature contains a report of a patient with a suprasellar arachnoid cyst who developed a spontaneous CSDH inside the cyst [10]. Our patient had a large sellar/suprasellar arachnoid cyst that did not contain blood, and his CSDH was localized on both cerebral convexities. Thus, it was improbable that the arachnoid cyst represented our patient’s etiology for the bilateral CSDH. He also did not have a history of head trauma. The only unusual activity performed in the preceding months was using a whole-body vibration machine.

## Conclusions

This case report demonstrates that using a whole-body vibration machine may lead to a chronic subdural hematoma. Due to the presence of cerebral atrophy in the elderly, a whole-body vibration machine should be used with caution. The formation of a chronic subdural hematoma can be associated with the presence of an arachnoid cyst; however, the sellar/suprasellar arachnoid cyst in our patient was unrelated to the formation of the hematoma.
